# TRMT13 inhibits the growth of papillary thyroid cancer by targeting ANAPC4: TRMT13-ANAPC4 ameliorates papillary thyroid cancer

**DOI:** 10.3724/abbs.2024010

**Published:** 2024-03-01

**Authors:** Lianyong Liu, Yan Wang, Mei Zou, Shiwei Chen, Fengying Wu, Xiangqi Li

**Affiliations:** 1 Department of Endocrinology and Metabolism Punan Hospital Shanghai 200125 China; 2 PharmaLegacy Laboratories Shanghai 201201 China; 3 Department of Intervention Gongli Hospital Naval Medical University Shanghai 200135 China; 4 Nursing Department Jinshan Branch of Shanghai Sixth People’s Hospital Shanghai 201599 China; 5 Department of Endocrinology and Metabolism Gongli Hospital Naval Medical University Shanghai 200135 China

**Keywords:** ANAPC4, CCDC76, CCDC family, methyltransferase, thyroid cancer, TRMT13

## Abstract

The recently discovered gene *TRMT13* encodes a type of RNA methylase and is a member of the *CCDC* family (also called CCDC76). Here, we delineate its role in papillary thyroid cancer (PTC). Bioinformatics analysis shows significant TRMT13 and ANAPC4 downregulation in PTC and reveals that the expression levels of both genes are linearly correlated. Subsequent analyses confirm that both TRMT13 and ANAPC4 expressions are downregulated in PTC tissues and that this change in expression has a significant impact on cancer diagnosis. We conduct assays on PTC cells subjected to *TRMT13* and *ANAPC4* silencing or overexpression to assess the biological effects of these genes. We also perform rescue experiments to validate the regulatory effects of TRMT13 on ANAPC4. A nude mouse tumor model is used to evaluate the effects of TRMT13 and ANAPC4 on PTC tumorigenesis. TRMT13 expression is decreased in PTC tissues and cell lines and is positively correlated with that of ANAPC4. Cell assays reveal that TRMT13/ANAPC4 attenuates the malignancy of PTC cells by restraining cell proliferation, migration and invasion, while rescue experiments corroborate that *ANAPC4* is a downstream target of TRMT13. In the nude mouse xenograft model, both TRMT13 and ANAPC4 inhibit tumor growth, and TRMT13 and ANAPC4 expression levels are significantly associated with survival. Taken together, these findings lead to the conclusion that TRMT13 inhibits PTC growth via ANAPC4, indicating a new role of TRMT13 and providing insights into the tRNA methyltransferase and coiled-coil domain-containing protein families.

## Introduction

As of 2020, there were 586,202 new cases of papillary thyroid cancer (PTC) worldwide. PTC accounts for 3.0% of all cancers and is the 11 ^th^ most common cancer [1]. In China, the incidence of PTC is approximately 15 cases per 100,000 person-years [2], and the five-year survival rate is approximately 84.3% [3]. Despite the relatively low malignancy of PTC, it is inoperable in approximately 5% of all locally advanced cases, and its rate of local recurrence is high [4]. For these reasons, the mortality rate of this disease is also elevated [5].

TRMT13, also known as coiled-coil domain containing 76 (CCDC76), was recently discovered. Liu *et al*. [6] reported that cytoplasmic hTrmt13 catalyzes the 2′- *O*-methylation of tRNAs, whereas nuclear hTrmt13 directly binds to DNA as a transcriptional coactivator. However, these functions are mutually exclusive. ANAPC4 or APC4 is an E3 ubiquitin ligase that regulates mitosis and the G1 phase [ 7‒ 10] . ANAPC4 abnormalities promote proliferation and correlate with tumor diameter in oral squamous cell carcinoma [11]. ANAPC4 inhibition favors docetaxel resistance in breast cancer [12]. ANAPC4 enables esophageal cancer and neural stem cells to proliferate [ 13, 14] . *ANAPC4* transcription and expression are regulated by tRNA methylation and affect cell proliferation [15]. To the best of our knowledge, however, the roles of ANAPC4 in PTC and its relationship with TRMT13 have not yet been investigated. Bioinformatics analysis of The Cancer Genome Atlas (TCGA) data revealed that both TRMT13 and ANAPC4 levels are significantly downregulated and that their expression levels are linearly correlated in PTC tissues [16]. Hence, we investigated the functions and interactions of TRMT13 and ANAPC4 in the onset and progression of PTC.

The present study aimed to clarify the biological roles of TRMT13 and ANAPC4 in PTC via cell ( *in vitro*) and nude mouse ( *in vivo*) models and to examine the regulatory effects of TRMT13 on ANAPC4. The discoveries made herein could help improve both the diagnosis and treatment of PTC and provide novel insights into the molecular roles of tRNA methyltransferases and coiled-coil domain-containing proteins.

## Materials and Methods

### Bioinformatics analysis

The Gene Expression Profiling Interactive Analysis (GEPIA) database ( http://gepia.cancer-pku.cn/) was used to examine thyroid cancer (THCA) cases. The GEPIA database is a new interactive web server that uses a standard pipeline to analyze RNA sequencing expression data for 9736 tumors and 8587 normal samples from the TCGA and the GTEx projects. The analysis included 512 tumor and 337 normal tissue samples.

### Clinical samples

Samples of papillary thyroid cancer (PTC) and adjacent normal tissues 2 cm from the tumors were collected from 30 patients at Punan Hospital (Shanghai, China). A pathologist microscopically examined the cells for the presence or absence of cancer cells. The patient inclusion criteria included (1) age range, 25–60 years; (2) pathologically confirmed PTC diagnosis; and (3) provided written informed consent. The patient exclusion criteria included (1) prior treatment for PTC; (2) co-infection; or (3) the presence of other malignant tumors. Survival time was calculated from the date of diagnosis to the date of the last visit or death. The median transfer RNA methyltransferase 13 homolog (TRMT13) or anaphase-promoting complex subunit 4 (ANAPC4) expression level was used as the threshold to assign the patients to the “high” and “low” expression groups. Receiver operating characteristic (ROC) was plotted to test the the area under the receiver operating curve (AUC) between PTC tumor and adjacent normal tissues. The research protocol was approved and the trial was supervised by the Ethics Committee of Punan Hospital.

### Cell culture

The human normal thyroid cell line HTori-3 and the PTC cell lines TTA1, ACT-1, KTC-1, TPC-1, KMH-2, and CAL-62 (Mlbio, Shanghai, China) were cultured in Dulbecco’s modified Eagle’s medium (DMEM) supplemented with 10% (v/v) fetal bovine serum (FBS; Sigma-Aldrich, St Louis, USA). The cells were maintained in a 5% CO _2_ incubator at 37°C and 95% relative humidity (RH), authenticated by the short tandem repeat (STR) method, and confirmed to be mycoplasma free.

### Cell transfection

TTA1 and CAL-62 cells expressing *TRMT13* or *ANAPC4* were transfected so that the aforementioned genes would be silenced or overexpressed, respectively. Group 1 included the control, TRMT13 ^+/+^, ANAPC4 ^+/+^, TRMT13 siRNA, ANAPC4 siRNA, plasmid-NC, and siRNA-NC. Group 2 included the control, TRMT13 ^+/+^, TRMT13 ^+/+^+ANAPC4 siRNA, TRMT13 siRNA, and TRMT13 siRNA+ANAPC4 ^+/+^. The plasmids were constructed by amplifying the *TRMT13*/ *ANAPC4* sequences via polymerase chain reaction (PCR) and cloning them into pIRES2-EGFP plasmids. Lentiviral transduction was performed to overexpress *TRMT13*/ *ANAPC4*. Small interfering RNAs (siRNAs) were designed to target *TRMT13*/ *ANAPC4* sequences and were transfected into the cells with Lipofectamine ^TM^ RNAiMAX (Thermo Fisher Scientific, Waltham, USA). The sequences of siRNAs were shown as follows: *ANAPC4* sense: GAGGCUCCAGUUUCCUGUAUGCAUU, antisense: AAUGCAUACAGGAAACUGGAGCCUC; and *TRMT13* sense: CAGGAUGGUGGUGGCCGCAGGGAAA, antisense: UUUCCCUGCGGCCACCACCAUCCUG. siRNA-NC was purchase from Qiagen (AllStars Negative Control siRNA; Qiagen, Hilden, Germany).

### RT-qPCR analysis

Cell and tissue RNAs were extracted using Trizol Reagent (Thermo Fisher Scientific) and reverse-transcribed into cDNA using High Capacity cDNA Reverse Transcription kit (Applied Biosystems, Foster City, USA). The *TRMT13* and *ANAPC4* mRNA expression levels were measured with SYBR Green-based qPCR [17] and normalized to that of glyceraldehyde 3-phosphate dehydrogenase ( *GAPDH*) using the 2 ^‒ΔΔCt^ method. The primers used were as follows: *TRMT13* forward primer: GCTGCCAGTTTTGAGGAAAG, reverse primer: TGCCCACATAATGTCTCCAA; and *ANAPC4* forward primer: CACCCCCTAACACAGAAGGA, reverse primer: CTGGCTTTTGCAAACACTGA.

### Western blot analysis

The total proteins were isolated from the cells and tissues with radioimmunoprecipitation assay (RIPA) buffer (Sigma-Aldrich), separated by sodium dodecyl sulfate-polyacrylamide gel electrophoresis (SDS-PAGE), and transferred to polyvinylidene fluoride (PVDF) membranes. The membranes were blocked with 5% skim milk and then incubated with rabbit primary antibodies against TRMT13 (1/5000; Abcam, Cambridge, USA) or ANAPC4 (2 μg/mL; Abcam). The membranes were then washed with phosphate-buffered saline (PBS; Sigma-Aldrich), followed by incubation with HRP-conjugated secondary antibody (ab205718, 1/5000; Abcam). Finally, the protein bands were visualized with an enhanced chemiluminescence (ECL) kit (Amersham Pharmacia Biotech, Piscataway, USA).

### Cell counting kit-8 (CCK-8) assay

One hundred microliters of cell suspension (5×10 ^4^/mL) was added to each well of a 96-well plate (Beyotime, Shanghai, China). Ten microliters of CCK-8 solution (Beyotime) was added to each well after 24, 48, and 72 h. The plates were mixed on an orbital shaker at 37°C for 1 min to ensure uniform dispersion of the cells and reagents. The plate was then incubated for 2 h, and the OD _450_ value of each well was subsequently measured with a microplate reader (LEx808, No. 25-315S; Lonza Group, Walkersville, USA).

### Wound healing assay

The cells were cultured in 6-well plates (Beyotime) under the aforementioned conditions until they reached 90%–95% confluence. A 200-μL pipette tip (Beyotime) was dragged from the top to the bottom of the well to create vertical scratches. The scratch width was initially measured, the cell debris were removed, and the remaining cells were cultured in serum-free medium (Sigma-Aldrich). Images of the cultures were captured 24 h later with a microscope (Hitachi, Tokyo, Japan) to evaluate wound healing.

### Colony formation assay

Two hundred cells were digested and resuspended under the aforementioned conditions for 14 days and then evenly distributed and cultured in medium. The latter was replaced every 2–3 days to ensure optimal cell growth. Macroscopic colony formation was observed, and the supernatant was discarded. The colonies were washed twice with PBS, fixed in 4% (v/v) paraformaldehyde (PFA; Sigma-Aldrich) for 15 min, stained with Giemsa (Sigma-Aldrich) for 20 min, washed again with PBS and air-dried. A gridded transparent film was placed on the plate and inverted to enumerate colonies composed of >20 cells.

### Transwell assay

The upper chamber of a 24-well Transwell ^®^ plate (Sigma-Aldrich) was coated with Matrigel ^®^ (1:8; v/v; Corning Life Sciences, Corning, USA). Cells were starved in serum-free medium at 37°C for 24 h, followed by digestion with trypsin. Then, 100 μL of cell suspension (5×10 ^4^ cells /mL) was added to the upper Transwell chamber, and 600 μL complete medium containing 20% (v/v) fetal bovine serum (FBS) was added to the lower chamber. After 24 h of incubation, the non-invading cells were washed away with PBS. The cells that infiltrated to the lower chamber were stained with 0.1% (w/v) crystal violet (Sigma-Aldrich) at 25°C for 20 min and fixed with 95% (v/v) ethanol. The number of cells was counted in five randomly selected fields under 400× magnification under a microscope.

### Xenotransplantation assay

Male BALB/C nude mice aged 4 weeks (Charles River Laboratories, Beijing, China) were used to assess tumorigenesis. The animals were housed at 24±1°C and 60%±5% RH. Group 1 ( *n*=6) included the control, TRMT13 ^+/+^, lentiviral NC, TRMT13 siRNA, and siRNA NC treatments. Group 2 ( *n*=6) included the control, ANAPC4 ^+/+^, lentiviral NC, ANAPC4 siRNA, and siRNA NC treatments. Group 3 ( *n*=6) included the control, TRMT13 ^+/+^, TRMT13 ^+/+^+ANAPC4 siRNA, TRMT13 siRNA, and TRMT13 siRNA+ANAPC4 ^+/+^ treatments. Five million cells overexpressing *TRMT13* or lentiviral NC, *TRMT13*-silenced, or siRNA NC; overexpressing *ANAPC4* or lentiviral NC; or *ANAPC4*-silenced or siRNA NC were prepared by transfection as above described. The lentivirus infection efficiency was verified by RT-qPCR. The transfected cells were resuspended in 200 μL of PBS and administered as a single injection into the axillary region of each nude mouse. Tumor formation at the injection site was observed on day 7, and body weight and tumor volume were measured every 4 days for the next 20 days. The animals were euthanized via CO _2_ inhalation when their tumor diameters were over 2 cm, after which the tumors were excised and weighed. A pathologist examined the tissue sections under a microscope to confirm xenograft thyroid tumor establishment. All animal experiment procedures used was approved by the Ethical Committee of Punan Hospital.

### Statistical analysis

All the experiments were conducted independently in triplicate. Data are expressed as the mean±standard deviations (SD) and subjected to analysis of variance (ANOVA) and Tukey’s multiple comparisons tests using GraphPad v. 7.0 (GraphPad Software, La Jolla, USA). Overall survival (OS) curves were plotted by the K-M method, and statistical significance was determined by the log-rank test. *P*<0.05 was considered statistically significant.

## Results

### TRMT13 and ANAPC4 expressions are downregulated in PTC tissues and their expressions are correlated with cancer diagnosis

**Figure 1:**
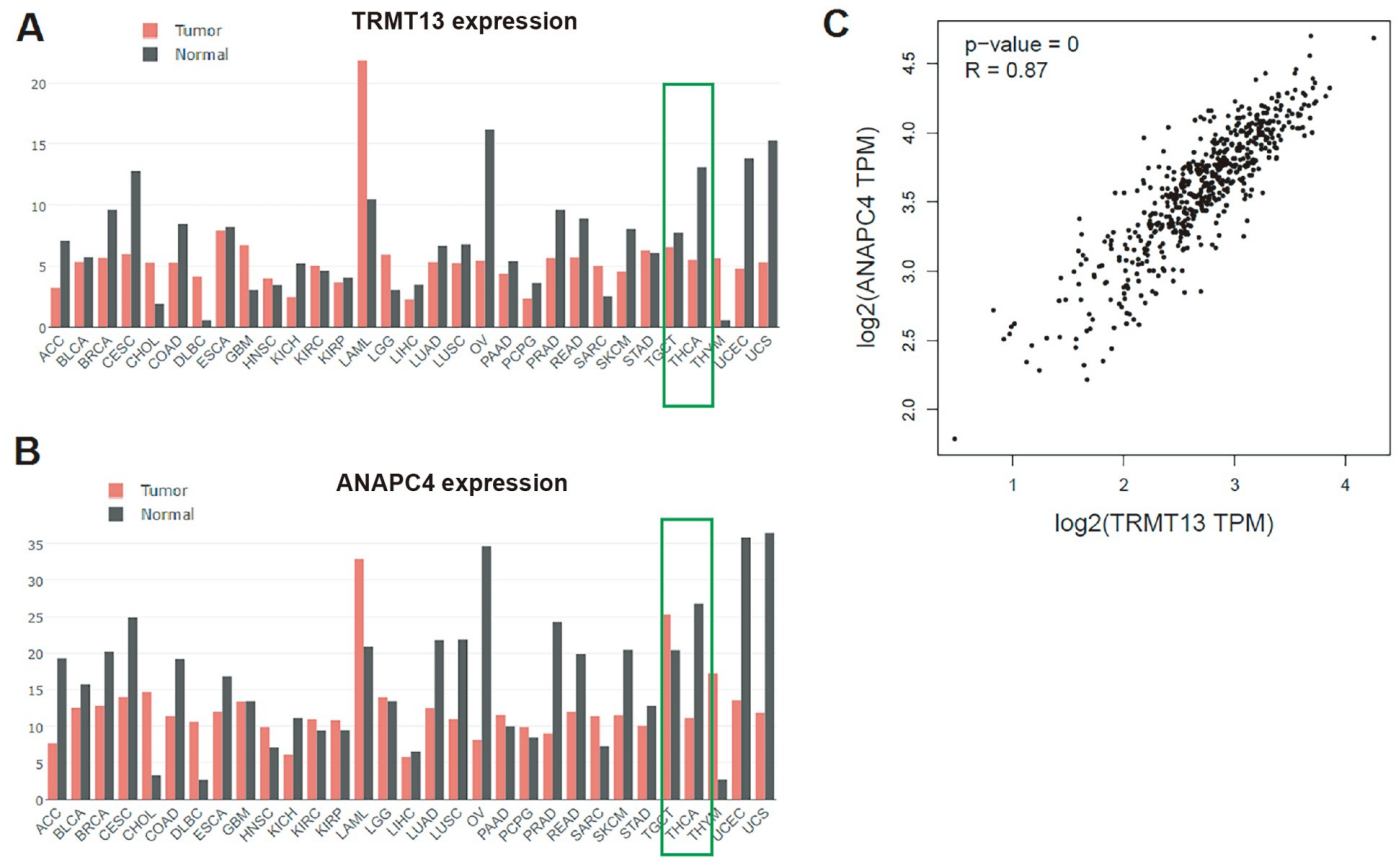
Figure 1 *TRMT13* and *ANAPC4* expressions are downregulated in PTC tissues (A) TRMT13 expression was altered in THCA tissues from the GEPIA database. (B) ANAPC4 expression was altered in THCA tissues from the GEPIA database. (C) Correlation between TRMT13 and ANAPC4 expressions in THCA and thyroid tissues from the GEPIA database. The analysis included 512 tumor and 337 normal tissue samples. THCA: thyroid carcinoma.

**Figure 2:**
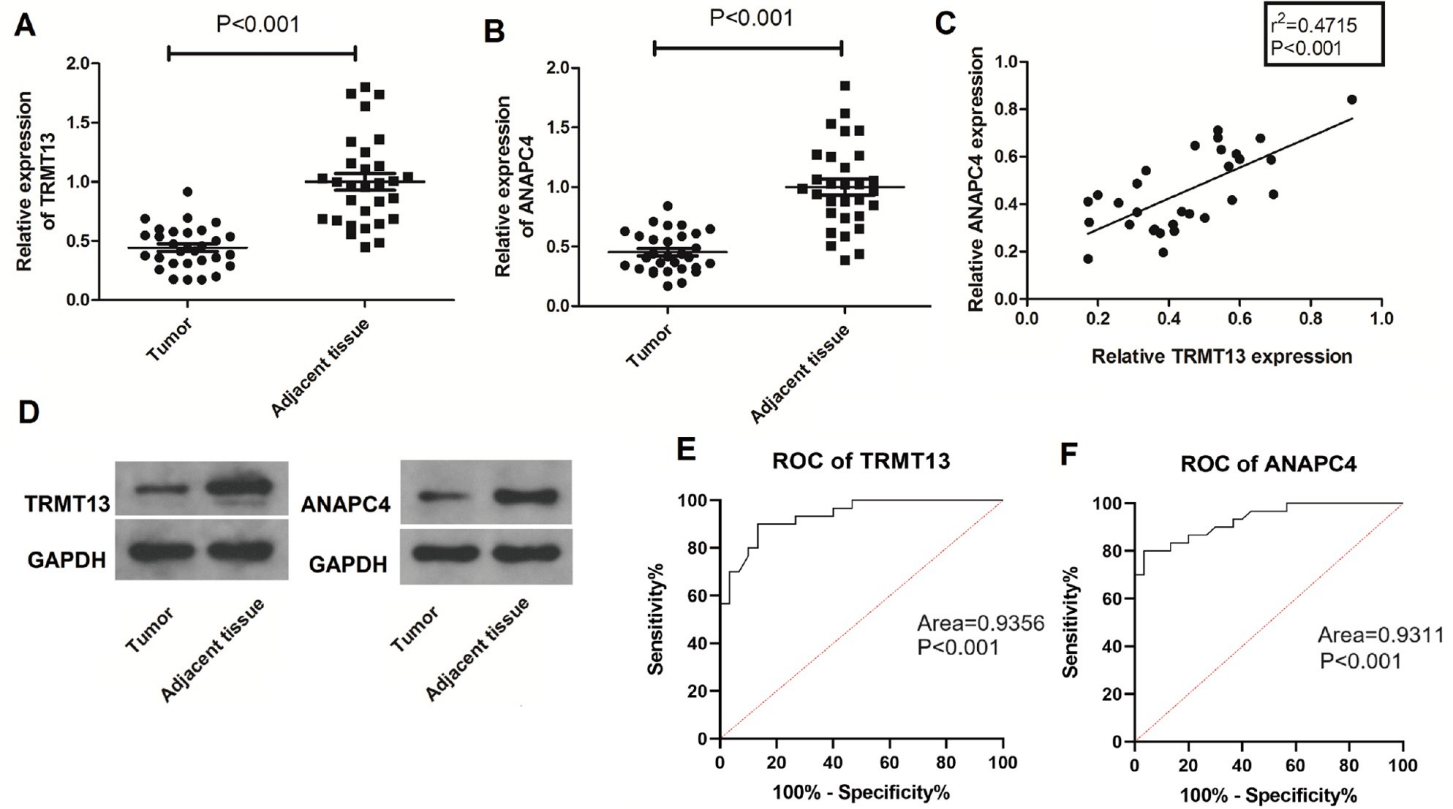
Figure 2 *TRMT13* and *ANAPC4* expressions are downregulated in PTC tissues and related with cancer diagnosis (A,B) TRMT13 and ANAPC4 expressions in PTC and adjacent normal tissues were measured by RT-qPCR ( n=30). (C) Correlation analysis of TRMT13 and ANAPC4 expressions in PTC tissues ( n=30). (D) TRMT13 and ANAPC4 protein expression levels in PTC and adjacent normal tissues were measured by western blot analysis ( n=30). (E,F) Receiver operating characteristic (ROC) analysis of TRMT13 and ANAPC4 expressions from PTC tumor from normal controls ( n=30).

The GEPIA database includes The Cancer Genome Atlas (TCGA; https://www.cancer.gov/ccg/research/genome-sequencing/tcga) data and reveals that both *TRMT13* and *ANAPC4* expressions are downregulated in PTC relative to normal tissues ( Figure 1A,B). The expression levels of these genes are significantly linearly correlated ( *P*=0; R=0.87) ( Figure 1C). The *TRMT13* and *ANAPC4* mRNA levels are significantly lower in the clinical PTC specimens than in the normal tissue specimens ( Figure 2A,B). The *TRMT13* and *ANAPC4* mRNA levels are positively correlated in the PTC tissue samples (r ^2^=0.4715; *P*<0.001) ( Figure 2C). The TRMT13 and ANAPC4 protein levels are lower in the PTC tissues than in the normal tissues ( Figure 2D). The ROC analysis revealed that *TRMT13* and *ANAPC4* expressions accurately distinguished PTC from normal controls ( Figure 2E,F).

### TRMT13 and ANAPC4 expressions are downregulated in PTC cells, which inhibits cell proliferation

**Figure 3:**
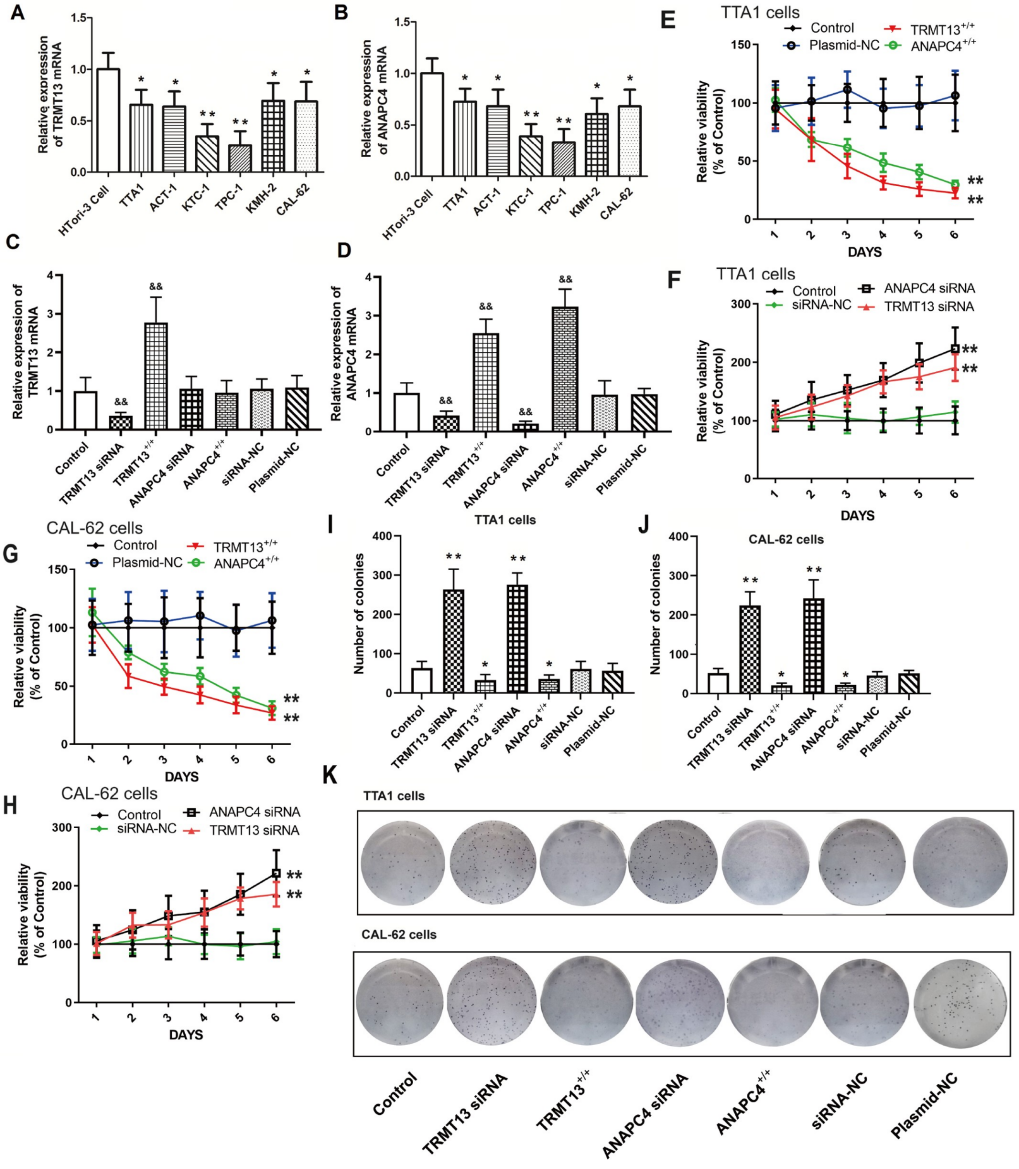
Figure 3 *TRMT13* and *ANAPC4* expressions are downregulated in PTC cells, which inhibits cell proliferation (A,B) TRMT13 and ANAPC4 mRNA levels in normal HTori-3 thyroid and PTC cells. TRMT13- and ANAPC4-silenced/overexpressing PTC cells were transfected and assigned to the control, TRMT13 +/+, ANAPC4 +/+, TRMT13 siRNA, ANAPC4 siRNA, plasmid-NC, or siRNA-NC groups. (C,D) TRMT13/ ANAPC4 mRNA levels in cells after plasmid or siRNA treatment. (E–H) Effects of TRMT13 and ANAPC4 on PTC cell proliferation. (I,J) Effects of TRMT13 and ANAPC4 on PTC cell colony formation. (K) Representative images of PTC cell colony formation. * P<0.05, ** P<0.01 vs the HTori-3 or control group. && P<0.01 vs Control.

**Figure 4:**
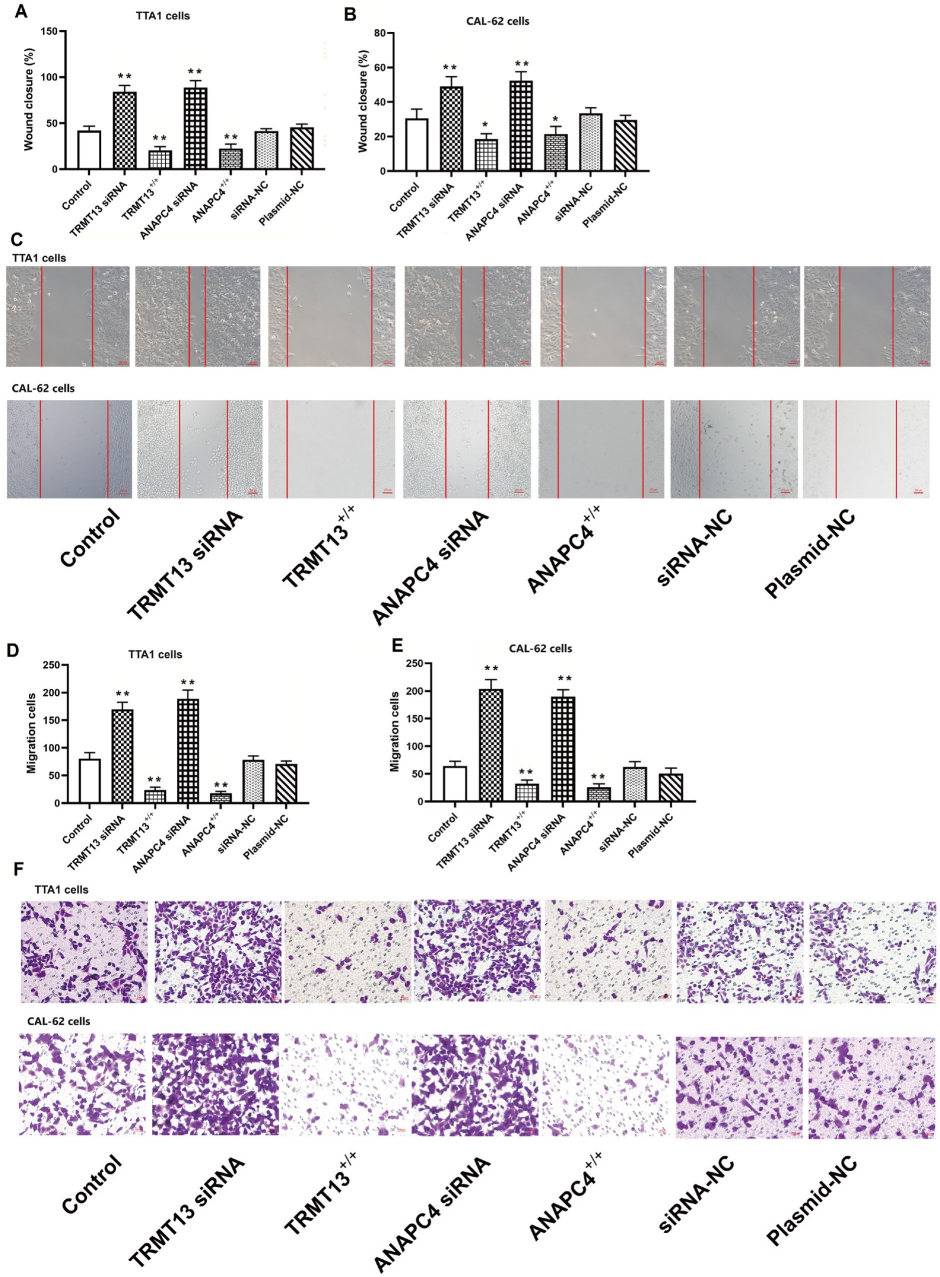
Figure 4 TRMT13 and ANAPC4 inhibit PTC cell migration and invasion TRMT13- and ANAPC4-silenced/overexpressing PTC cells were transfected and assigned to the control, TRMT13 +/+, ANAPC4 +/+, TRMT13 siRNA, ANAPC4 siRNA, plasmid-NC, or siRNA-NC groups. (A,B) Effects of TRMT13 and ANAPC4 on cancer cell migration. (C) Representative images of the PTC cell scratch healing assay results. (D,E) Effects of TRMT13 and ANAPC4 on PTC cell invasion. (F) Representative images of the Transwell assay of PTC cells. ** P<0.01 vs Control.

The *TRMT13* and *ANAPC4* expression levels were significantly lower in all six PTC cell lines than those in the normal HTori-3 cell line ( Figure 3A,B). The *TRMT13* and *ANAPC4* mRNA levels after plasmid exposure or siRNA treatment are shown in Figure 3C,D. Transfection with the TRMT13 plasmid or siRNA significantly altered the *TRMT13* mRNA level relative to that in the untreated cells. However, neither the ANAPC4 plasmid nor the siRNA treatment changed the *TRMT13* mRNA level. In contrast, the TRMT13 plasmid and siRNA treatments significantly modified the *ANAPC4* mRNA level. We generated cells in which *TRMT13* and *ANAPC4* were overexpressed or silenced to investigate the effects of these genes and their products. The results showed that *TRMT13* or *ANAPC4* upregulation inhibited the proliferation of TTA1 and CAL-62 cells, whereas *TRMT13* or *ANAPC4* downregulation promoted the proliferation of TTA1 and CAL-62 cells ( Figure 3E–H). We then assessed the impact of *TRMT13* and *ANAPC4* on PTC malignancy and found that upregulation of either or both of these genes inhibited colony formation of TTA1 and CAL-62 cells ( Figure 3I–K). Compared with those in the control group, cancer cell migration and invasion were lower in the TRMT13 ^+/+^ and ANAPC4 ^+/+^ groups but greater in the TRMT13 siRNA and ANAPC4 siRNA groups ( Figure 4).

### TRMT13 and ANAPC4 inhibit PTC progression *in vivo*

**Figure 5:**
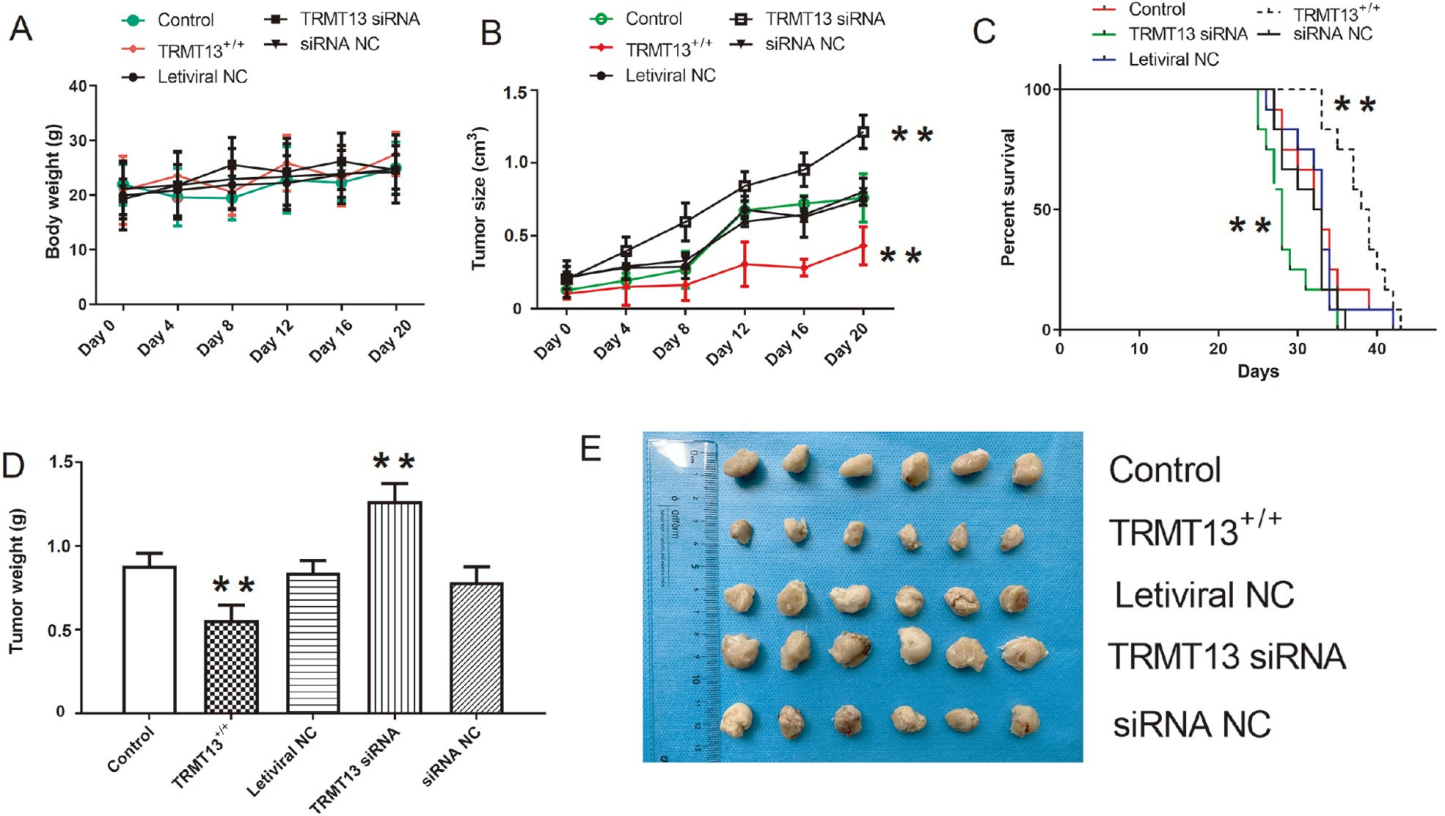
Figure 5 TRMT13 inhibits PTC progression *in vivo* Cells from the control, TRMT13 +/+, lentiviral NC, TRMT13 siRNA, and siRNA NC groups were used to construct xenograft tumor-bearing nude mouse models. (A) Effect of TRMT13 on body weight. (B) Effect of TRMT13 on tumor size. (C) Effect of TRMT13 on survival. (D) Effect of TRMT13 on tumor weight. (E) Tumors were excised from mice of each group. ** P<0.01 vs Control.

We created tumor-bearing nude mouse models from the control, TRMT13 ^+/+^, lentiviral NC, TRMT13 siRNA, and siRNA NC groups to investigate the impact of *TRMT13* on PTC progression *in vivo*. Body weight did not significantly change within groups during tumor formation or growth ( *P*>0.05; Figure 5A). However, *TRMT13* overexpression significantly reduced tumor volume, whereas *TRMT13* silencing significantly promoted tumor growth and reduced survival ( Figure 5B,C). The TRMT13 siRNA and TRMT13 ^+/+^ groups presented larger and smaller mean tumor masses, respectively, than the control group ( Figure 5D). Figure 5E showed the inhibitory effects of *TRMT13* on PTC progression *in vivo*.

**Figure 6:**
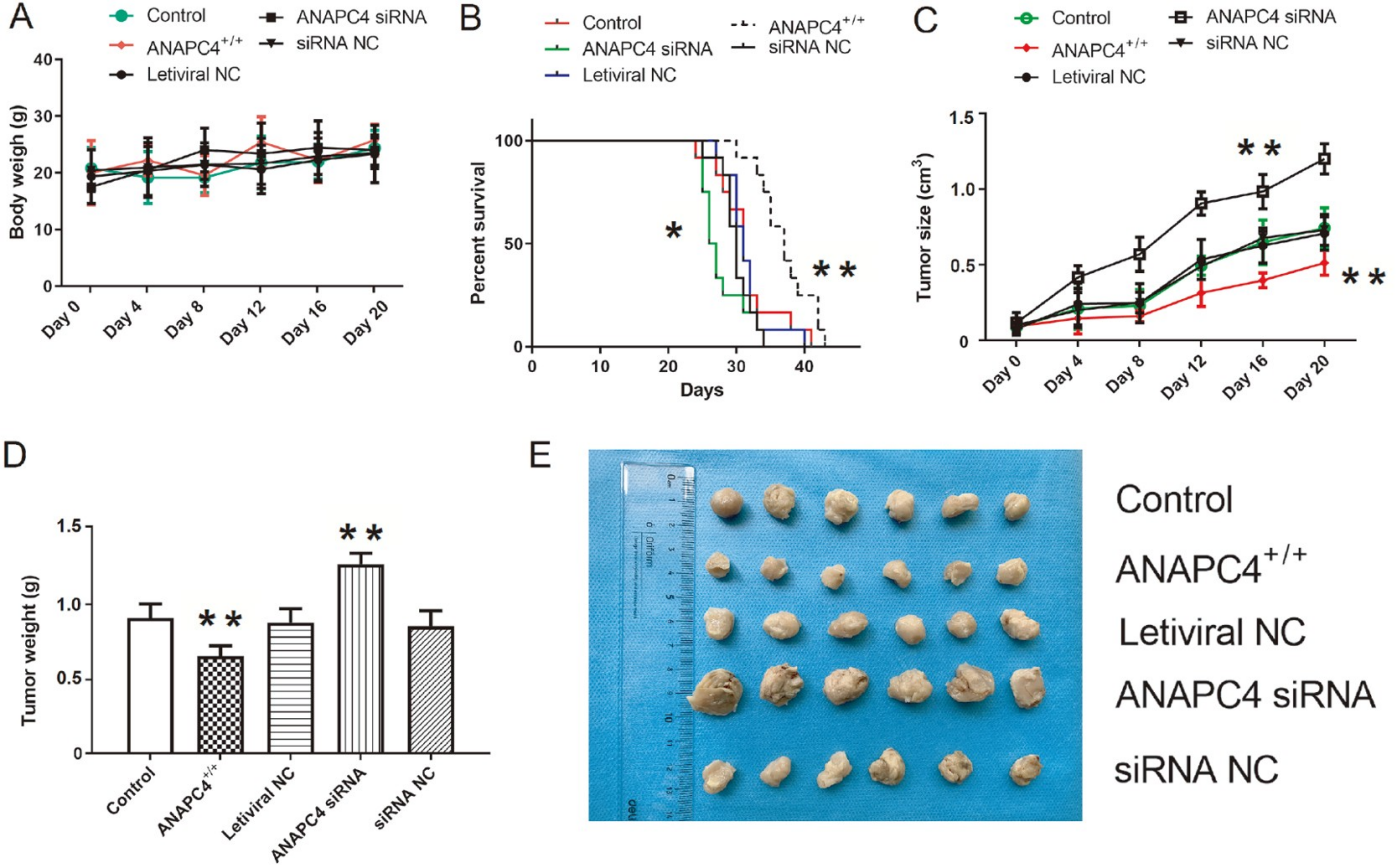
Figure 6 ANAPC4 inhibits PTC progression *in vivo* Cells from the control, ANAPC4 +/+, lentiviral NC, ANAPC4 siRNA, and siRNA NC groups were used to construct xenograft tumor-bearing nude mouse models. (A) Effect of ANAPC4 on body weight. (B) Effect of ANAPC4 on tumor size. (C) Effect of ANAPC4 on survival. (D) Effect of ANAPC4 on tumor weight. (E) Tumors were excised from mice of each group. * P<0.05, ** P<0.01 vs Control.

We constructed xenotransplantation models using cells from the control, ANAPC4 ^+/+^, lentiviral NC, ANAPC4 siRNA, and siRNA NC groups to investigate the effects of *ANAPC4* on PTC progression *in vivo*. Body weight did not significantly change within groups during tumor formation or growth ( *P*>0.05; Figure 6A). However, *ANAPC4* overexpression significantly reduced tumor volume, whereas *ANAPC4* silencing significantly promoted tumor growth and reduced survival ( Figure 6B,C). The ANAPC4 siRNA and ANAPC4 ^+/+^ groups presented larger and smaller mean tumor masses, respectively, than the control group ( Figure 6D). Figure 6E showed the inhibitory effects of ANAPC4 on PTC progression *in vivo*.

### ANAPC4 reverses the effects of *TRMT13 silencing* on PTC cells

**Figure 7:**
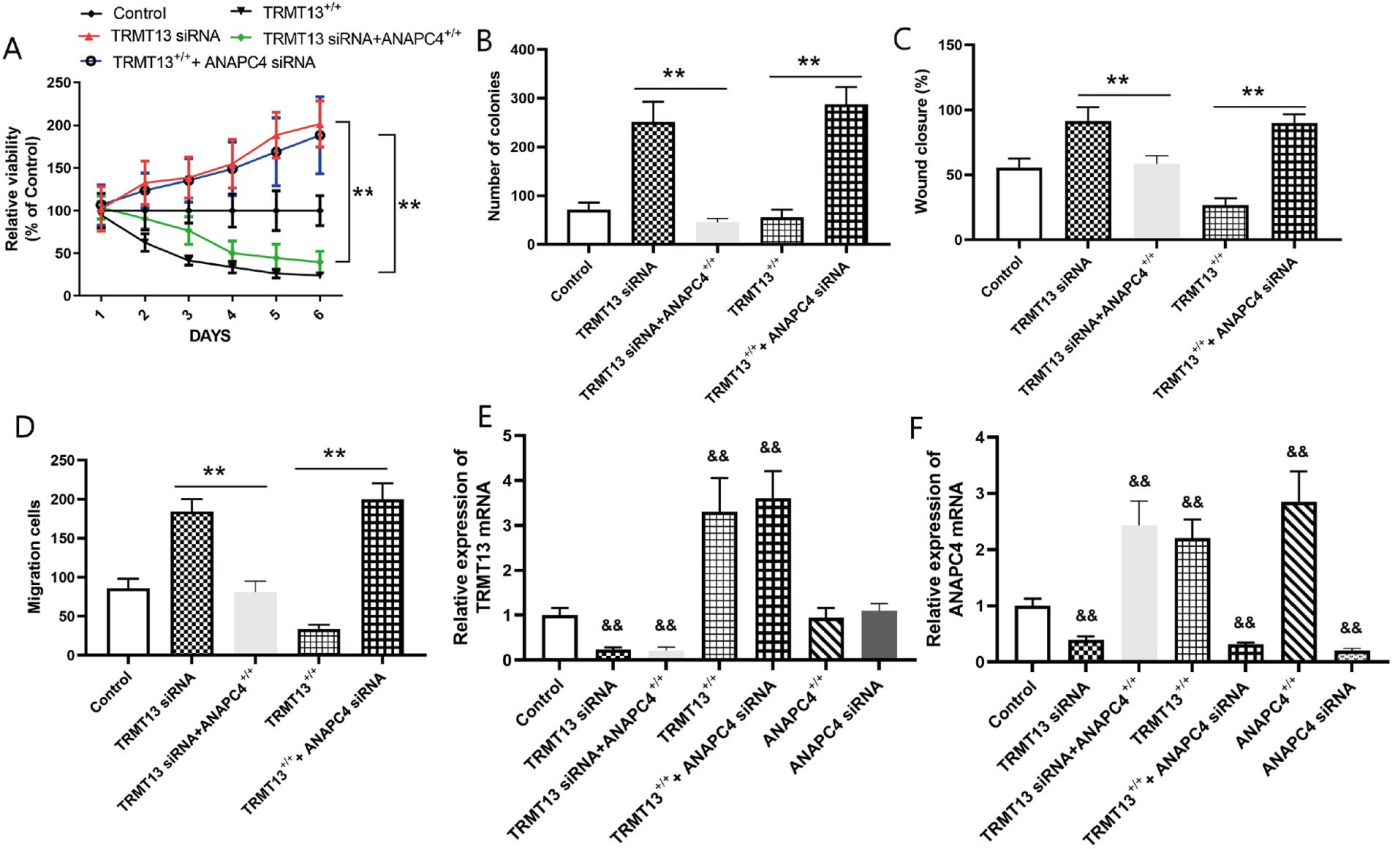
Figure 7 ANAPC4 reverses the effects of TRMT13 on PTC cells ANAPC4 was silenced when TRMT13 was overexpressed and vice versa. PTC cells were assigned to the control, TRMT13 +/+, TRMT13 +/++ANAPC4 siRNA, TRMT13 siRNA, or TRMT13 siRNA+ANAPC4 +/+ group. (A) Effect of TRMT13 overexpression/ ANAPC4 silencing and TRMT13 silencing/ ANAPC4 overexpression on PTC cell proliferation. (B) Effect of TRMT13 overexpression/ ANAPC4 silencing and TRMT13 silencing/ ANAPC4 overexpression on PTC cell colony formation. (C,D) Effects of TRMT13 overexpression/ ANAPC4 silencing and TRMT13 silencing/ ANAPC4 overexpression on PTC cell migration and invasion. (E) Relative TRMT13 expression in cells subjected to TRMT13 overexpression/ ANAPC4 silencing and TRMT13 silencing/ ANAPC4 overexpression. (F) Relative TRMT13 expression in cells subjected to ANAPC4 overexpression/ ANAPC4 silencing and TRMT13 silencing/ ANAPC4 overexpression. ** P<0.01 between groups; && P<0.01 vs Control.

We silenced *ANAPC4* combined with overexpressing TRMT13 or silenced *TRMT13* combined with overexpressing ANAPC4 to investigate the involvement of ANAPC4 in the mechanism by which TRMT13 regulates PTC progression. We assigned the PTC cells to the control, TRMT13 ^+/+^, TRMT13 ^+/+^+ANAPC4 siRNA, TRMT13 siRNA, and TRMT13 siRNA+ANAPC4 ^+/+^ groups. The results demonstrated that silencing of *ANAPC4* reversed the inhibition of PTC cell proliferation and colony formation caused by *TRMT13* overexpression, while *ANAPC4* overexpression impeded PTC progression by silencing *TRMT13* ( Figure 7A,B). Moreover, cell migration and invasion were significantly greater in the TRMT13 ^+/+^+ANAPC4 siRNA group than in the TRMT13 ^+/+^ group. In contrast, cell migration and invasion were significantly lower in the TRMT13 siRNA+ANAPC4 ^+/+^ group than in the TRMT13 siRNA group ( Figure 7C,D). These findings suggest that ANAPC4 contributes to the inhibitory effect of TRMT13 on PTC. We then assigned the PTC cells to the control, TRMT13 ^+/+^, TRMT13 ^+/+^+ANAPC4 siRNA, TRMT13 siRNA, TRMT13 siRNA+ANAPC4 ^+/+^, ANAPC4 ^+/+^, and ANAPC4 siRNA groups and measured their TRMT13 and ANAPC4 mRNA levels. Figure 7E,F showed that the TRMT13 plasmid and siRNA treatments significantly altered the TRMT13 mRNA level, while the ANAPC4 plasmid and siRNA treatments did not. In contrast, TRMT13 plasmid and siRNA treatment significantly altered ANAPC4 mRNA level.

### ANAPC4 reverses the effects of *TRMT13* silencing on PTC progression *in vivo*

**Figure 8:**
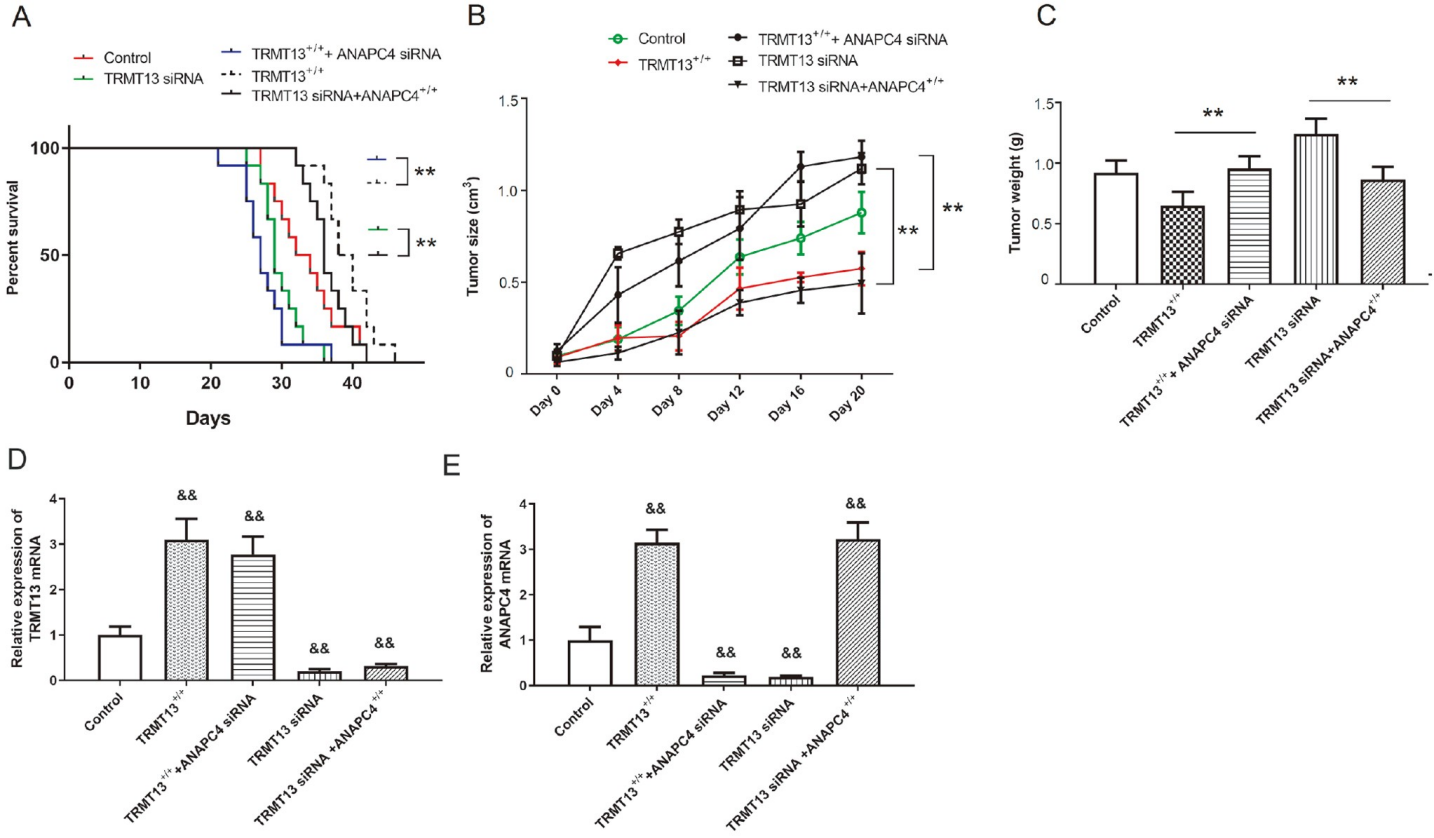
Figure 8 ANAPC4 reverses the effects of TRMT13 on PTC progression *in vivo* Cells from the control, TRMT13 +/+, TRMT13 +/++ANAPC4 siRNA, TRMT13 siRNA, and TRMT13 siRNA+ANAPC4 +/+ groups were used to construct xenograft tumor-bearing nude mouse models. (A) Effect of TRMT13 overexpression/ ANAPC4 silencing and TRMT13 silencing/ ANAPC4 overexpression on survival. (B) Effect of TRMT13 overexpression/ ANAPC4 silencing and TRMT13 silencing/ ANAPC4 overexpression on tumor size. (C) Effect of TRMT13 overexpression/ ANAPC4 silencing and TRMT13 silencing/ ANAPC4 overexpression on tumor weight. (D) Relative TRMT13 expression in tumor tissues from cells subjected to TRMT13 overexpression/ ANAPC4 silencing and TRMT13 silencing/ ANAPC4 overexpression. (E) Relative TRMT13 expression in tumor tissue from cells subjected to ANAPC4 overexpression/ ANAPC4 silencing and TRMT13 silencing/ ANAPC4 overexpression. ** P<0.01 between groups. && P<0.01 vs Control.

We then constructed xenograft models by injecting nude mice with cells from the control, TRMT13 ^+/+^, TRMT13 ^+/+^+ANAPC4 siRNA, TRMT13 siRNA, or TRMT13 siRNA+ANAPC4 ^+/+^ groups. *TRMT13* overexpression and silencing improved and worsened survival, respectively, whereas *ANAPC4* silencing counteracted the improvement in survival afforded by TRMT13 upregulation. While TRMT13 downregulation worsened mouse survival, ANAPC4 overexpression improved survival ( Figure 8A). Tumor volume and mass were greater in the TRMT13 ^+/+^+ANAPC4 siRNA group than in the TRMT13 ^+/+^ group and lower in the TRMT13 siRNA+ANAPC4 ^+/+^ group than in the TRMT13 siRNA group ( Figure 8B,C). The relative TRMT13 and ANAPC4 expression levels in the tumor tissue are shown in Figure 8D,E. While the *TRMT13* plasmid and siRNA treatments significantly altered TRMT13 mRNA level, the *ANAPC4* plasmid and siRNA treatments had no significant effect on *TRMT13* mRNA level. In contrast, the *TRMT13* plasmid and siRNA treatment significantly altered *ANAPC4* mRNA level.

## Discussion

Dysregulated gene expression is characteristic of all malignancies and neoplasms, including PTC. According to GeneCards ( https://www.genecards.org/), TRMT13 is implicated in tRNA methylation. The tRNA methyltransferase family has been extensively studied. METTL1-mediated m7G tRNA modification enhances lenvatinib resistance in hepatocellular carcinoma (HCC) [18]. The N7-methylguanosine (m7G) tRNA modification may modulate autophagy in certain cancers [19]. Methyltransferase-like protein 8-dependent mitochondrial tRNA m3C modification mediates the epitranscriptomic regulation of cortical neurogenesis [20].

*TRMT13* ( *CCDC76*) belongs to a large, extensively studied gene family. CCDC50-mediated autophagy regulates NLRP3 inflammasome activation [21]. HPV-CCDC106 integration promotes cervical cancer progression [22]. To the best of our knowledge, however, the biological function of TRMT13 in PTC has not been reported, and there is limited information on its relationship with cancer onset and progression. The *TRMT13* gene ( *CCDC76*) was first described in a study by Weinmann *et al*. [23]. The authors performed a wide-ranging chromatin immunoprecipitation (ChIP) assay targeting the αE2F transcription factor (TF) and selected 68 targets, including *CCDC76* [23]. In terms of size and domain architecture, hCCDC76 resembles scTrm13 except that the latter has two estimated zinc finger domains, whereas the former has only one [24]. Liu *et al*. [6] reported that hTrmt13 catalyzes 2′- *O*-methylation in tRNAs and, in contrast, coactivates transcription [6]. These findings suggest that RNA-modifying enzymes may also have noncatalytic functions.

Here, bioinformatics analyses revealed that TRMT13 is downregulated in PTC and that its expression is inversely correlated with cancer cell growth/tumor progression ( Figure 1). qPCR and western blot analysis demonstrated that *TRMT13* was downregulated in clinical cancer tissue specimens. ROC analyses indicated that TRMT13 expression significantly influenced patient cancer diagnosis. Hence, TRMT13 might suppress PTC tumors. We also employed *TRMT13*-silenced and *TRMT13*-overexpressing PTC cells to confirm the effects of these treatments on PTC cell malignancy ( Figure 3). Treatment with the *TRMT13* plasmid or siRNA significantly altered TRMT13 mRNA level, whereas treatment with the *ANAPC4* plasmid or siRNA did not. However, treatment with the *TRMT13* plasmid or siRNA significantly modified ANAPC4 mRNA levels. Thus, *TRMT13* is upstream of *ANAPC4* in the signaling pathway.

Experiments in nude mice demonstrated that TRMT13 significantly suppressed PTC tumors *in vivo* ( Figure 5). Thus, TRMT13 may participate in PTC onset and progression. However, these findings contradict those reported by Liu *et al*. [6], who identified hTrmt13 as a breast and cervical cancer oncogene. This apparent discrepancy may be explained by the functional differences in genes among various types of cancer. ANAPC4 is a component of the anaphase-promoting complex/cyclosome (APC/C), which regulates the cell cycle by targeting specific proteins for degradation. APC/C dysregulation has been implicated in different malignancies and neoplasms. Bradfield *et al*. [25] reported that ANAPC4 expression was significantly associated with favorable prognosis in patients with endometrioid cancer. siRNA screening revealed that ANAPC4 is involved in chromosomal instability tolerance in diploid cells [26]. *ANAPC4* inactivation confers resistance to multiple TTK protein kinase inhibitors in triple-negative breast cancer [27]. Here, bioinformatics analysis revealed that ANAPC4 is significantly downregulated in PTC cells ( Figure 1) and tissues. ROC analysis demonstrated that ANAPC4 significantly influences cancer diagnosis. Therefore, ANAPC4 may suppress PTC tumor onset and progression.

To the best of our knowledge, however, there is no known direct relationship between *TRMT13* and *ANAPC4* expression. qPCR analysis indicated that *ANAPC4* expression was significantly lower in PTC tissue than in normal tissue and was positively correlated with *TRMT13* expression. Here, *ANAPC4* overexpression inhibited PTC cell growth, while *ANAPC4* silencing promoted PTC cell growth and tumor growth ( Figures 3 and 6). These findings suggest that *ANAPC4* is involved in the molecular mechanism by which TRMT13 inhibits PTC onset and progression. Rescue experiments confirmed that TRMT13 modulates ANAPC4 expression ( Figures 7 and 8). Hence, ANAPC4 partially mediates the inhibitory effect of TRMT13 on PTC. The present work provides preliminary evidence that *TRMT13*/ *ANAPC4* suppresses PTC onset and progression. However, further research is needed to determine the clinical significance of these genes in this type of cancer by investigating the associations between gene alterations and patient outcomes or treatment responses and between gene expression profiles and patient outcomes. Moreover, future research will focus on the regulatory mechanism between TRMT13 and ANAPC4.

In summary, the present work established that TRMT13 and ANAPC4 suppress PTC cell proliferation, migration, and invasion *in vitro* and reduce PTC tumors *in vivo*. They also impeded tumor growth in a nude mouse PTC xenograft model. The findings presented herein lay the foundation for future research on the clinical application of TRMT13 and ANAPC4 in the diagnosis and treatment of PTC. This study also facilitates the clarification of the roles of tRNA methyltransferases and coiled-coil domain-containing proteins.
